# Zonula occludens toxins and their prophages in *Campylobacter* species

**DOI:** 10.1186/s13099-016-0125-1

**Published:** 2016-09-15

**Authors:** Fang Liu, Hoyul Lee, Ruiting Lan, Li Zhang

**Affiliations:** School of Biotechnology and Biomolecular Sciences, University of New South Wales, Kensington, Sydney, 2052 Australia

**Keywords:** *Campylobacter concisus*, Zonula occludens toxin (Zot), *Campylobacter*, Prophage

## Abstract

**Background:**

We previously showed that zonula occludens toxin (Zot) encoded by *Campylobacter concisus zot*^808T^ gene has the potential to initiate inflammatory bowel disease. This Zot protein caused prolonged intestinal epithelial barrier damage, induced intestinal epithelial and macrophage production of tumor necrosis factor-α and enhanced the responses of macrophages to other microbes. In order to understand the potential virulence of Zot proteins in other *Campylobacter* species, in this study we examined their presence, similarities, motifs and prophages.

**Methods:**

The presence of Zot proteins in *Campylobacter* species was examined by searching for the Zot family domain in multiple protein databases. Walker A and Walker B motifs in Zot proteins were identified using protein sequence alignment. A phylogenetic tree based on *Campylobacter zot* genes was constructed using maximum-likelihood method. *Campylobacter* Zot proteins were compared using protein sequence alignment. The *zot*-containing prophages in *Campylobacter* species were identified and compared with known prophage proteins and other viral proteins using protein sequence alignment and protein BLAST.

**Results:**

Twelve Zot proteins were found in nine *Campylobacter* species/subspecies. Among these *Campylobacter* species, three species had two Zot proteins and the remaining six species/subspecies had one Zot protein. Walker A and Walker B motifs and a transmembrane domain were found in all identified *Campylobacter* Zot proteins. The twelve *Campylobacter zot* genes from the nine *Campylobacter* species/subspecies formed two clusters. The Zot_CampyType_1_ proteins encoded by Cluster 1 *Campylobacter zot* genes showed high similarities to each other. However, Zot_CampyType_2_ proteins encoded by Cluster 2 *Campylobacter zot* genes were more diverse. Furthermore, the *zot*-containing *Campylobacter* prophages were identified.

**Conclusion:**

This study reports the identification of two types of *Campylobacter* Zot proteins. The high similarities of Zot_CampyType_1_ proteins suggest that they are likely to have similar virulence. Zot_CampyType_2_ proteins are less similar to each other and their virulent properties, if any, remain to be examined individually.

**Electronic supplementary material:**

The online version of this article (doi:10.1186/s13099-016-0125-1) contains supplementary material, which is available to authorized users.

## Background

*Campylobacter concisus* is a Gram-negative spiral shaped motile bacterium [1]. Their growth under both anaerobic and microaerobic conditions is largely determined by the presence of H_2_ [2]. This bacterium usually colonizes the human oral cavity [3, 4]. However, it may also colonize the intestinal tract of some individuals and its prevalence in intestinal biopsies has been associated with human inflammatory bowel disease (IBD) [5–9]. Translocation of *C. concisus* from the oral cavity to intestinal tract has been suggested to be a cause of a subgroup of IBD [10]. *Campylobacter concisus* has also been suggested to be involved in diarrheal disease due to the frequent isolation of this bacterium from diarrheal stool samples [8, 11–13].

*Campylobacter concisus* zonula occludens toxin (Zot) is encoded by the *zot* gene located in CON_phi2 prophage [14, 15]. The *zot* genes in different *C. concisus* strains have polymorphisms [14]. In a recent study, we examined the effects of *C. concisus* Zot encoded by *zot*^808T^ gene on human intestinal epithelial cells and macrophages using cell line models. In that study, we found that *C. concisus* Zot caused prolonged intestinal epithelial barrier damage, induced intestinal epithelial and macrophage production of proinflammatory cytokines such as tumor necrosis factor-α, and enhanced the responses of macrophages to *Escherichia coli* [16]. These data suggest that *C. concisus* Zot may play a role in initiating chronic intestinal inflammatory conditions such as IBD through damaging the intestinal barrier and enhancing the immune responses to luminal microbes.

In addition to *C. concisus*, four additional *Campylobacter* species including *Campylobacter ureolyticus*, *Campylobacter corcagiensis*, *Campylobacter gracilis* and *Campylobacter iguaniorum* were recently reported to possess the *zot* gene [17–21]. The similarities of Zot proteins in different *Campylobacter* species have not been examined. In this study, we examined Zot proteins and their genes in *Campylobacter* species, which revealed two clusters of *Campylobacter zot* genes and two types of *Campylobacter* Zot proteins. Furthermore, we identified the prophages that contain *zot* genes in *Campylobacter* species.

## Methods

### Examination of the presence of Zot proteins in *Campylobacter* species

The presence of Zot proteins in *Campylobacter* species was examined by searching for proteins in *Campylobacter* species that have the Zot family domain in multiple protein databases including NCBI protein database, InterPro and Pfam [22–24]. The Zot proteins in these databases were annotated based on the presence of Zot family domain.

### Examination of the presence of Walker A and Walker B in *Campylobacter* Zot proteins

It is shown in the InterPro database that the Zot family proteins (InterPro entry identity: IPR008900) belong to p-loop containing nucleoside triphosphate hydrolase (p-loop NTPase) superfamily. The proteins of p-loop NTPase superfamily have Walker A and Walker B motifs [25]. In this study, we examined the presence of Walker A and Walker B motifs in *Campylobacter* Zot proteins by protein alignment using Clustal Omega software [26]. The Walker A motif has a sequence of GxxxxGK[S/T], where x is any residue and the Walker B motif has a sequence of hhhh[D/E], where h is a hydrophobic residue [25].

### Generation of a phylogenetic tree based on *zot* genes in *Campylobacter* species

To examine the genetic relationship between the *zot* genes in different *Campylobacter* species, the nucleotide sequences of the *zot* genes in *Campylobacter* species identified above were obtained from NCBI genome database. These sequences were used to generate a phylogenetic tree using the maximum likelihood method implemented in molecular evolutionary genetics analysis software version 6.0 [27]. In order to differentiate the *zot* genes in different *Campylobacter* species, the *zot* genes found in different *Campylobacter* species were indicated by the last four digits of their locus tag numbers.

### Comparison of *zot*-containing prophages in *Campylobacter* species

To examine whether the *zot* genes in different *Campylobacter* species are carried by prophages similar to that in *C. concisus*, the *zot*-containing prophages in these *Campylobacter* species were identified by examination of the genes adjacent to their *zot* genes. The prophages were defined based on the presence of integrase, hypothetical proteins and attachment sites [28]. The attachment sites were identified by the presence of repetitive sequences located at both ends of the prophages [28].

The proteins in the identified *Campylobacter* prophages in this study were compared with the corresponding proteins in *C. concisus* prophages CON_phi2 and CON_phi3. The comparison was performed using Clustal Omega [26]. Proteins sharing more than 40 % sequence identity were considered to have high similarities and were recorded [29].

### Comparison of the proteins of *zot*-containing prophages in *Campylobacter* species with other viral proteins

To examine whether the *zot* containing prophages identified in *Campylobacter* species are similar to previously reported prophages, proteins of *Campylobacter* prophages were compared with viral proteins in the NCBI non-redundant protein sequence database (taxonomy identity for viruses: 10,239) using protein BLAST with default settings [22]. The identified viral proteins with E-values lower than 10 were noted. For prophage proteins which shared sequence similarities with multiple viral proteins, the viral proteins with the lowest E-values were noted. The full length sequences of the identified viral proteins were then aligned with the *Campylobacter* prophage proteins using Clustal Omega [26]. The protein identities were calculated by dividing the number of identical amino acids by the total number of amino acids in proteins from *Campylobacter* prophages. The *Campylobacter* prophage proteins were also compared with viral proteins in the virus pathogen database and analysis resource (ViPR) using BLAST with a cut-off E-value of 10. In addition to *C. concisus*, the *zot* gene was also found in other bacterial species such as *Vibrio cholerae* and *Neisseria meningitis* [30, 31]. The identities between *Campylobacter* Zot, *V. cholerae* Zot and *N. meningitidis* Zot proteins were also compared in this study.

### Prediction of secreted proteins and transmembrane proteins in *Campylobacter* prophages

Secreted proteins in *zot*-containing prophages in different *Campylobacter* species were predicted using SignalP 4.1 and SecretomeP 2.0. The software SignalP 4.1 predicts secreted proteins based on the presence of the signal peptide at the N-terminus [32]. The software SecretomeP 2.0 predicts non-classical (not signal peptide triggered) protein secretion based on analysis of post-translational and localizational aspects of the proteins [33]. Transmembrane proteins were predicted using the software Phobius [34].

## Results

### The Zot proteins in different *Campylobacter* species and their Walker A and Walker B motifs

Twelve Zot proteins were found in nine *Campylobacter* species/subspecies. Three *Campylobacter* species including *C. concisus*, *C. ureolyticus* and *C. corcagiensis* had two Zot proteins and the remaining six *Campylobacter* species/subspecies including *C. gracilis*, *Campylobacter jejuni* subsp. *doylei*, *Campylobacter jejuni* subsp. *jejuni*, *Campylobacter hyointestinalis* subsp. *hyointestinalis*, *C. hyointestinalis* subsp. *lawsonii* and *C. iguaniorum* had one Zot protein (Table 1).

**Table 1:** Zot proteins in *Campylobacter* species/subspecies

*Campylobacter* species/subspecies	Strain	Number of Zot proteins	Locus tag	Source of isolation	Reference
*C. concisus*	13826	2	CCC13826_2276 CCC13826_0191	Human faeces	Gb0000058^a^
*C. ureolyticus*	DSM 20703	2	C512_RS0103935 C512_RS0100745	Human amniotic fluid	[35]
*C. corcagiensis*	CIT045	2	BG71_RS0106485 BG71_RS0104620	Lion-tailed macaques faeces	[36]
*C. gracilis*	RM3268	1	CAMGR0001_2456	Human oral cavity	Gb0003988^a^
*C. jejuni* ssp*. doylei*	269.97	1	JJD26997_0348	Human blood	Gb0000076^a^
*C. jejuni* ssp*. jejuni*	60004	1	CJE11_RS08060	Chicken	SAMN02429007^@^
*C. hyointestinalis* ssp*. hyointestinalis*	DSM 19053	1	CR67_01870	Porcine intestine	SAMN01176354^@^
*C. hyointestinalis* ssp. *lawsonii*	CCUG 27631	1	CHL_RS06765	Porcine intestine	[37]
*C. iguaniorum*	RM11343	1	CIG11343_RS03950	Alpaca faeces	[21]

^a^Genome online database project ID. ^@^NCBI Biosample ID

**Fig. 1:**
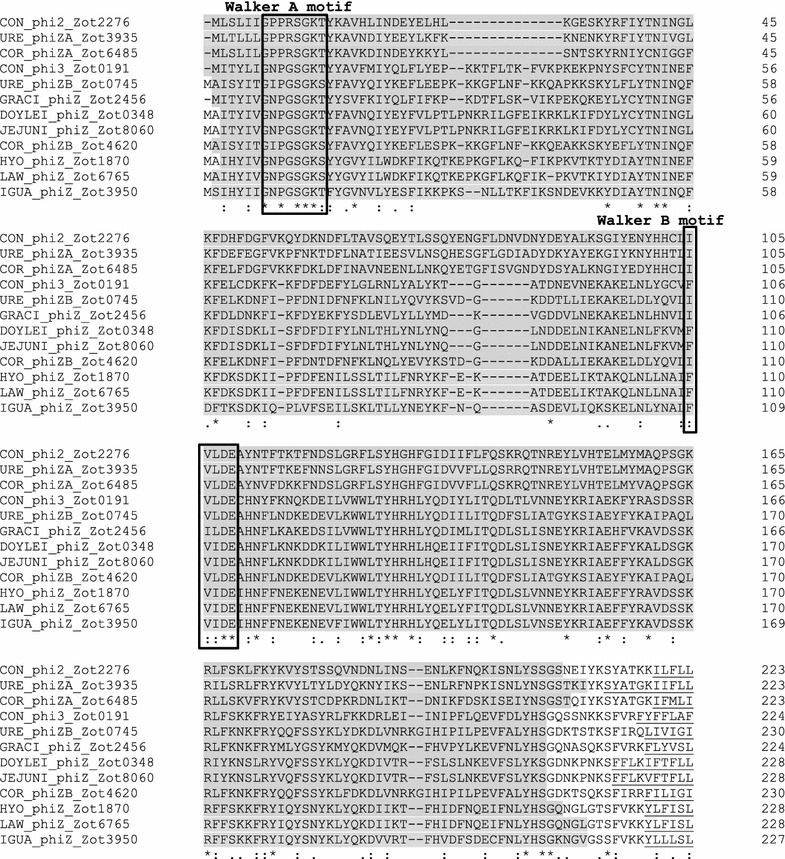
Walker A and walker B motifs in *Campylobacter* Zot proteins. *Campylobacter* Zot proteins have a transmembrane domain (*underlined*). The amino acids prior to the transmembrane domains constitute the Zot family domains (approximately 1–210, *shaded in grey*) in different *Campylobacter* Zot. The Zot family domains belong to p-loop NTPase superfamily. Walker A and walker B motifs are in the N-terminus of *Campylobacter* Zot proteins. Walker A has a sequence of GxxxxGK[S/T], where x is any residue. Walker B motif has a sequence of hhhh[D/E], where h is a hydrophobic residue [25]

The Zot family domains were localized at the N-terminal side of the identified *Campylobacter* Zot proteins, prior to the transmembrane domains. The Zot family proteins had p-loop NTPase domains and the entry identity for p-loop NTPase domain was IPR027417 in InterPro database. We identified the Walker A and Walker B motifs in *Campylobacter* Zot proteins, which were at the N-terminal side of the Zot proteins (Fig. 1).

### The phylogenetic tree formed based on *zot* genes in *Campylobacter* species

**Fig. 2:**
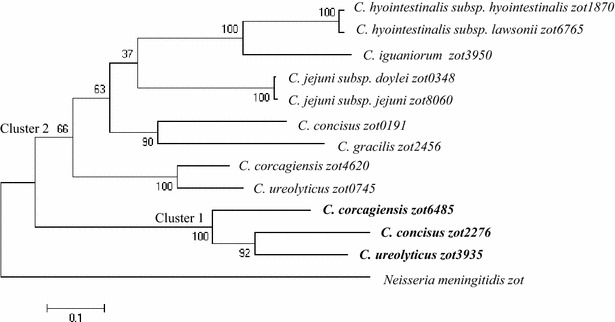
The phylogenetic tree generated based on *zot* genes in different *Campylobacter* species. Maximum likelihood method was used to generate the phylogenetic tree. *Bootstrap* values were generated from 1000 replicates. Cluster 1 *zot* genes are shown in *bold*. The *zot* gene from *Neisseria meningitides* (strain 69166) was used as the outgroup

The *Campylobacter zot* genes formed two clusters. Cluster 1 contained three *zot* genes, including *C. concisus zot2276, C. ureolyticus zot3935 and C. corcagiensis zot6485.* Cluster 2 contained nine *Campylobacter zot* genes (Fig. 2).

### Comparison of Zot_CampyType_1_ and Zot_CampyType_2_ proteins

**Fig. 3:**
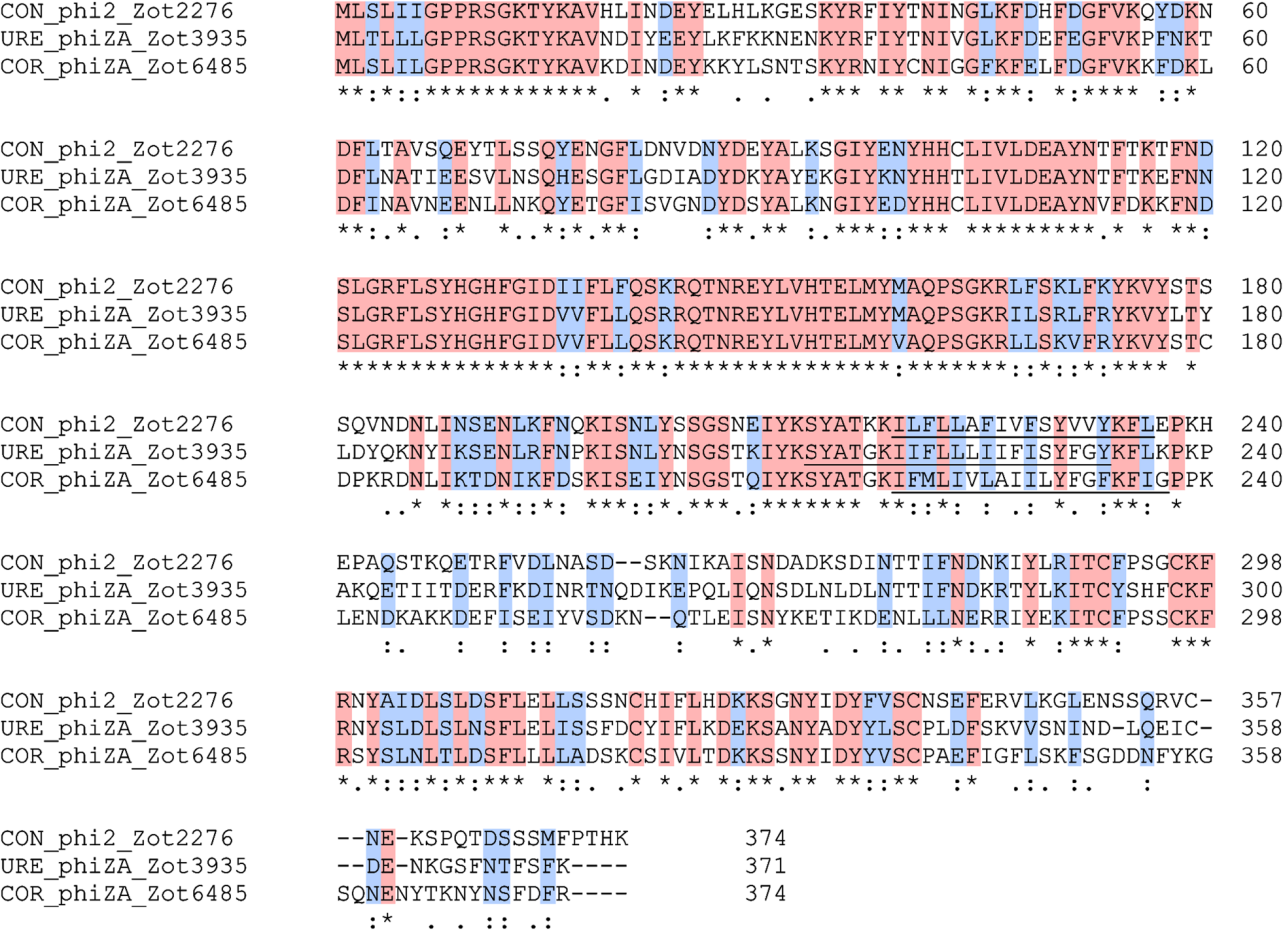
Comparison of Zot_CampyType_1_ proteins. The protein similarities were compared using Clustal Omega. *Asterisk* indicates identical amino acids (*shaded in red*). *Colon* indicates conservative mutations (*shaded in blue*). *Dot* indicates semi-conservative mutations. Transmembrane domains are *underlined*

**Fig. 4:**
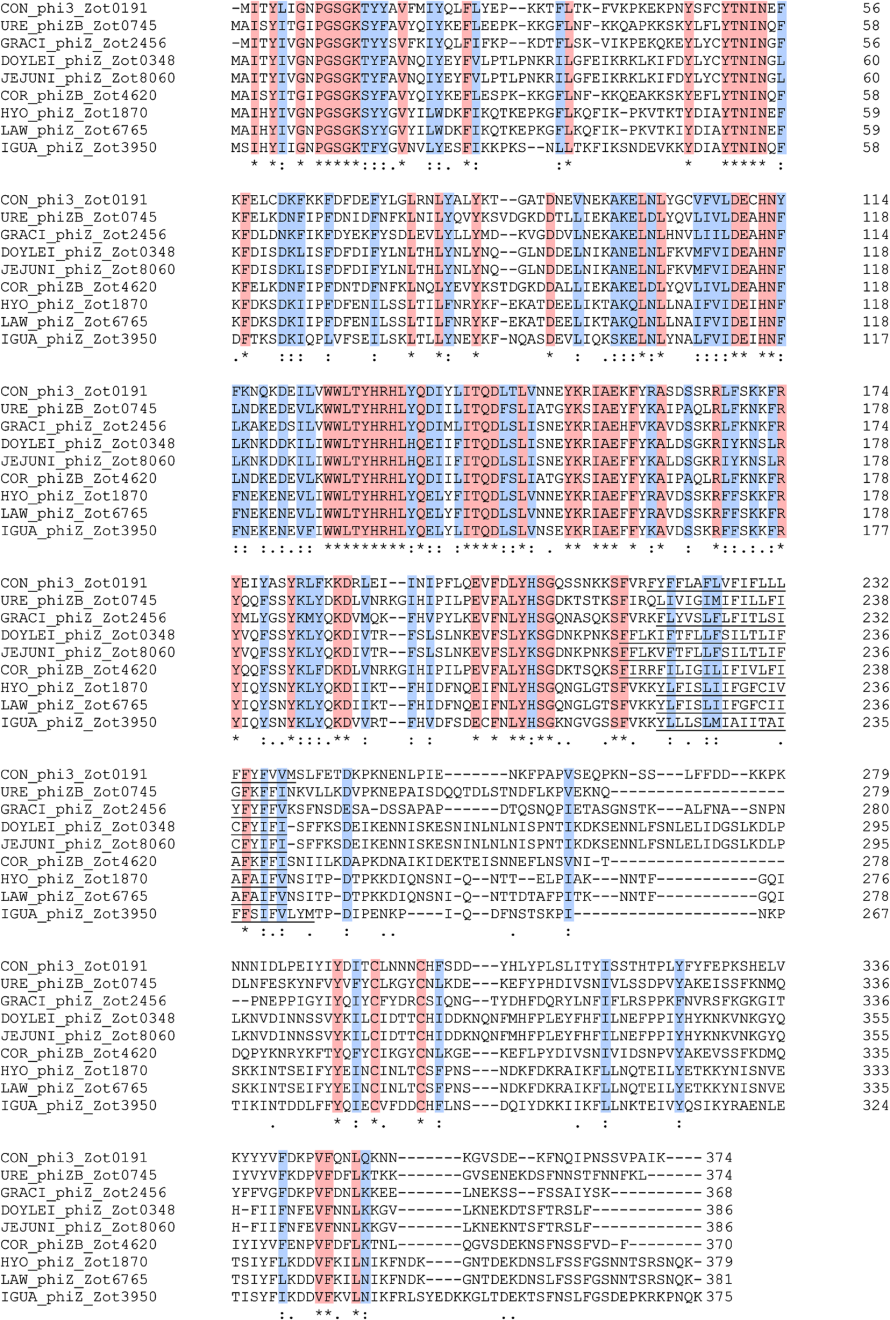
Comparison of Zot_CampyType_2_ proteins. The protein similarities were compared using Clustal Omega. *Asterisk* indicates identical amino acids (*shaded in red*). *Colon* indicates conservative mutations (*shaded in blue*). *Dot* indicates semi-conservative mutations. Transmembrane domains are *underlined*

The Zot proteins encoded by Cluster 1 and Cluster 2 *Campylobacter zot* genes were referred to as Zot_CampyType_1_ and Zot_CampyType_2_ proteins respectively. The three Zot_CampyType_1_ proteins had high similarities; they shared 171 identical amino acids and 77 conservative mutations (Fig. 3). The Zot_CampyType_2_ proteins were less similar to each other as compared to Zot_CampyType_1_ proteins; they had 71 identical amino acids and 65 conservative mutations (Fig. 4).

### The *zot*-containing prophages in different *Campylobacter* species and their similarities to *C. concisus* prophages CON_phi2 or CON_phi3

**Fig. 5:**
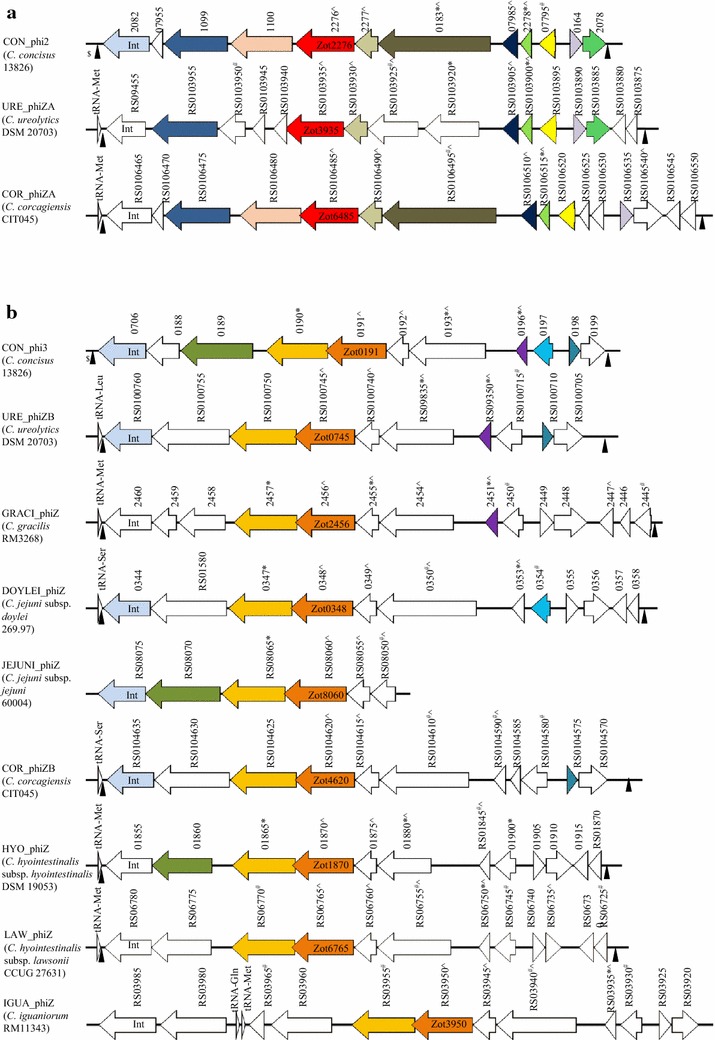
Schematic illustration of protein similarities in *zot*-containing *Campylobacter* prophages. **a** *Campylobacter* prophages containing Zot_CampyType_1_ proteins. **b** *Campylobacter* prophages containing Zot_CampyType_2_ proteins. The prophages and their host *Campylobacter* strains (*in bracket*) are listed at the left side of the figure. Proteins with more than 40 % identical amino acids with proteins in CON_phi2 or CON_phi3 were labeled with the same color. The numbers above the proteins are locus tags of the genes in the NCBI database. *Int* indicates integrase. *Asterisk* and *Hashtag* indicate proteins predicted to be secreted via classic secretory pathway or non-classic secretory pathway respectively. *Caret* indicates transmembrane proteins. *Dollar* indicates multiple insertion sites for CON_phi prophages in *C. concisus* 13826 in which only the first attachment site (for CON_phi1) overlapped with tRNA [15]. *Filled triangle* indicates attachment sites

We identified the *zot*-containing prophages in different *Campylobacter* species. Each of these prophages began with an integrase and had a number of hypothetical proteins (Fig. 5; Additional file 1). The attachment sites were found (Table 2). These prophages were inserted within either tRNA-Met or tRNA-Ser genes, except for URE_phiZB, which was inserted into tRNA-Leu gene (Table 2). For the prophage in *C. iguaniorum*, two tRNA genes were found after the integrase, suggesting that multiple insertions have occurred.

**Table 2:** The attachment sites of *zot*-containing *Campylobacter* prophages

Prophage	Start^a^	End^a^	Attachment gene sequence^b^	tRNA (locus_tag)
CON_phi1, CON_phi2 and CON_phi3 (*C. concisus* 13826)^c^	1582286	1582311	*TTCAAATCCCTCTCTGTCCGCCA*CCA	tRNA-Ser (CCC13826_RS07905)
	1587508	1587533	TTCAAATCCCTCTCTGTCCGCCACCA	
	1597113	1597138	TTCAAATCCCTCTCTGTCCGCCACCA	
	1606718	1606743	TTCAAATCCCTCTCTGTCCGCCACCA	
	1616160	1616185	TTCAAATCCCTCTCTGTCCGCCACCA	
CON_phi4 (*C. concisus* 13826)	946941	946993	*CTCATAACCCGAAGGTCGGCGGTTCAA* *ATCCGTCCTCCGCAACCA*AATACCGA	tRNA-Met (CCC13826_RS04780)
	937290	937342	CTCATAACCCGAAGGTCGGCGGTTCAA ATCCGTCCTCCGCAACCAAATACCGA	
URE_phiZA (*C. ureolyticus* DSM 20703)	68067	68122	*ATAACCCGAAGGTCGGAGGTTCAAGTCCT* *TCCTCTGCAACCA*AATCACCATTTTAC	tRNA-Met (C512_RS0103965)
	57811	57866	ATAACCCGAAGGTCGGAGGTTCAAGTCCT TCCTCTGCAACCAAATCACCATTTTAC	
URE_phiZB (*C. ureolyticus* DSM 20703)	139564	139610	*GTTCAAGTCTCGCTGATCGCACCA*TTA AAGAAAAAATTAAGAATACT	tRNA-Leu (C512_RS0100765)
	130103	130149	GTTCAAGTCTCGCTGATCGCACCATTA AAGAAAAAATTAAGAATACT	
COR_phiZA (*C. corcagiensis* CIT045)	254051	254088	*CGAAGGTCAGGGGTTCAAGTCCCTTCT* *CTGCAACCA*AA	tRNA-Met (SA94_RS06290)
	265302	265339	CGAAGGTCAGGGGTTCAAGTCCCTTCT CTGCAACCAAA	
COR_phiZB (*C. corcagiensis* CIT045)	257240	257264	*GTTCAAATCCCTCTCTGTCCGCCA*C	tRNA-Ser (SA94_RS04510)
	247319	247343	GTTCAAATCCCTCTCTGTCCGCCAC	
GRACI_phiZ (C*. gracilis* RM3268)	112826	112871	*CTCATAACCCGAAGGTCGGTGGTTCAA* *ATCCACCCTCTGCAACCAA*	tRNA-Met (CAMGR0001_2931)
	102518	102563	CTCATAACCCGAAGGTCGGTGGTTCAA ATCCACCCTCTGCAACCAA	
DOYLEI_phiZ (*C. jejuni* subsp. *doylei* 269.97)	303215	303241	*AGGGTTCAAATCCCTCTCTGTCCGCCA*	tRNA-Ser (JJD26997_RS01570)
	313165	313191	AGGGTTCAAATCCCTCTCTGTCCGCCA	
HYO_phiZ (*C. hyointestinalis* subsp. *hyointestinalis* DSM 19053)	360409	360446	*CTCATAACCCGAAGGTCGGAGGTTCAA* *GTCCTTCTCTC*	tRNA-Met (CR67_RS01810)
	369716	369753	CTCATAACCCGAAGGTCGGAGGTTCAA GTCCTTCTCTC	
HYO_phiZ (*C. hyointestinalis* subsp. *lawsonii* CCUG 27631)	1283134	1283238	*CTCATAACCCGAAGGTCGGAGGTTCAAGT* *CCTTCTCTCGCAACCA*AATAAGCATAAAA TCATCTTTTAAAGCACATTGTTTTAAAGCT TAAAATAATCTTACTTT	tRNA-Met (CHL_RS06785)
	1273720	1273824	CTCATAACCCGAAGGTCGGAGGTTCAAGT CCTTCTCTCGCAACCAAATAAGCATAAAA TCATCTTTTAAAGCACATTGTTTTAAAGCT TAAAATAATCTTACTTT	

^a^The start and end positions for the attachment sites refer to the nucleotide position within the contig containing the prophage genomes, except for *C. concisus* strains 13826, *C. jejuni* subsp. *doylei* 269.97 and *C. hyointestinalis* subsp. *lawsonii* CCUG 27631 which refer to the nucleotide position in the full genome

^b^Attachment sites overlapped with 3′ end of tRNA, the overlapped sequences were italic

^c^Multiple insertion sites for CON_phi prophages in *C. concisus* 13826 in which only the first attachment site (for CON_phi1) overlapped with tRNA. In NCBI database, the contig encoding JEJUNI_phiZ did not cover the full prophage genome; therefore it was unable to locate the attachment sites. No attachment site was identified in IGUA_phiZ

Prophages containing Zot_CampyType_1_ proteins had high similarities to CON_phi2. Eight proteins in URE_phiZA and nine proteins in COR_phiZA were found to have more than 40 % identities (41-73 %) with proteins in CON_phi2 (Fig. 5a; Additional file 1). Proteins in prophages containing Zot_CampyType_2_ proteins were more diverse. However, three to five proteins in these prophages had more than 40 % identities with that in CON_phi3 (40–72 %) (Fig. 5b; Additional file 1).

### The similarities between proteins in *Campylobacter* prophages and viral proteins

The *zot*-containing *Campylobacter* were compared with known viral proteins in NCBI non-redundant protein sequence database. The proteins within each individual prophage showed low similarities to multiple phage proteins, except for CCC13826_0188 in CON_phi3 that shared 43 % identity with a phage transferase from an uncultured phage (Additional file 2). The Zot proteins in *Campylobacter* prophages had low similarities with the Zot proteins in *V. cholerae* and *N. meningitidis* (15–21 %) (Additional files 3, 4). None of the *Campylobacter* prophage proteins shared significant similarities with viral proteins in ViPR database. These data suggest that the *zot*-containing prophages in *Campylobacter* species are new prophages that have not been previously characterized.

### Secreted and transmembrane proteins in *Campylobacter* prophages

Proteins secreted via both classical secretory pathway (with signal peptides) and non-classical secretory pathway (without signal peptides), as well as transmembrane proteins were found in all *Campylobacter* prophages (Fig. 5).

## Discussion

In addition to *C. concisus*, a number of other *Campylobacter* species were recently reported to have the *zot* genes [17–21]. In this study, we found that *Campylobacter zot* genes formed two clusters (Fig. 2). Most of the *Campylobacter zot* genes were in Cluster 2, and Cluster 1 contained only three *zot* genes. The three *Campylobacter* species that had Cluster 1 *zot* genes also contained Cluster 2 *zot* genes. The remaining six *Campylobacter* species/subspecies contained Cluster 2 z*ot* genes only. These data show that Cluster 2 *zot* gene is more prevalent in *Campylobacter* species as compared to Cluster 1 *zot* genes.

Zot_CampyType_1_ proteins, which were encoded by Cluster 1 *zot* genes, were highly similar to each other. However they were less similar to Zot_CampyType_2_ proteins that were encoded by Cluster 2 *Campylobacter zot* genes. The *zot* gene in CON_phi2 prophage in *C. concisus* belongs to Zot_CampyType_1_. Using cell culture models, we previously showed that Zot_CampyType_1_ in *C. concisus* encoded by *zot*^808T^ polymorphism damaged intestinal epithelial barrier by induction of epithelial apoptosis and induced production of proinflammatory cytokines such as TNF-α in HT-29 cells and THP-1 macrophage-like cells, supporting its role as a potential virulence factor [16]. The high similarities between Zot_CampyType_1_ proteins in the three different *Campylobacter* species suggest that they may have similar effects on human cells. Great variations in protein sequences between Zot_CampyType_1_ and Zot_CampyType_2_ proteins as well as within Zot_CampyType_2_ proteins were observed in this study. Given this, the effects of Zot_CampyType_2_ proteins on human cells, if any, require to be examined individually.

A transmembrane domain was found in all Zot proteins, showing that Zot proteins are transmembrane proteins. Furthermore, all Zot proteins contained Walker A and Walker B motifs, which are conserved motifs of p-loop NTPase superfamily [25]. P-loop NTPase bind to NTP typically ATP or GTP through the Walker A and B motifs, which are involved in diverse cellular functions [25]. Future studies should be conducted to examine whether *Campylobacter* Zot proteins have NTPase activities.

In this study, we identified a number of *zot*-containing prophages in other *Campylobacter* species in additional to previous reported prophages in *C. concisus* (Fig. 5). These prophages have an integrase, a number of hypothetical proteins and attachment sites (Fig. 5; Table 2; Additional file 1), which satisfy the previously defined criteria for prophages [28]. The proteins in individual *Campylobacter* prophages identified in this study have low similarities with multiple viral proteins, suggesting that they are new prophages that have not being characterized previously (Additional file 2).

*Campylobacter* Zot proteins had very low similarities to *V. cholerae* Zot and *N. meningitis* Zot proteins (Additional files 3, 4). These data showed that despite having a common name, the amino acid sequences of Zot proteins in different bacterial species vary greatly. Thus, they may not necessarily exhibit the same effects on human cells.

## Conclusions

This study reports the identification of two types of *Campylobacter* Zot proteins. The high similarities of Zot_CampyType_1_ proteins suggest that they are likely to have similar virulence. Zot_CampyType_2_ proteins were less similar to each other and their virulent properties, if any, remain to be examined individually. This study provides useful information for further examination of *Campylobacter* Zot proteins as potential virulence factors.

## Additional files

10.1186/s13099-016-0125-1 Protein identities between prophages in other *Campylobacter* species and *C. concisus* CON_phi2 and CON_phi3. Zot proteins are in bold. Integrase proteins are underlined #Identity: Percentage of identical amino acids (number of identical amino acids divided by number of amino acids in proteins from *C. concisus* 13826).
10.1186/s13099-016-0125-1 Comparison of *Campylobacter* prophage proteins with known viral proteins. #Identity: Percentage of identical amino acids (number of identical amino acids divided by number of amino acids in proteins from *Campylobacter* species).
10.1186/s13099-016-0125-1 Comparison of *Campylobacter* Zot proteins with *V. cholerae* Zot and *N. meningitidis* Zot. ^#^Identity: percentage of identical amino acids (number of identical amino acids. divided by number of amino acids of *V. cholerae* Zot or *N. meningitidis* Zot). *V. cholerae* Zot sequence Accession No. is AAF29547. *N. meningitidis* Zot sequence Accession No. is EJU63554.
10.1186/s13099-016-0125-1 Comparison of Zot proteins from *Campylobacter* species, *N. meningitidis* and *V. cholerae*. * indicates identical amino acids (shaded in red). : indicates conservative mutations (shaded in blue). .indicates semi-conservative mutations. Transmembrane domains are underlined. Walker A and walker B motifs in the N-terminus of *Campylobacter* Zot proteins were identified and boxed. Walker A has a sequence of GxxxxGK[S/T], where x is any residue. Walker B motif has a sequence of hhhh[D/E], where h is a hydrophobic residue [25].

## Abbreviations

IBD: inflammatory bowel disease
Zot: zonula occludens toxin
Zot_CampyType_1_: *Campylopbacter* Zot protein encoded by Cluster 1 *zot* gene
Zot_CampyType_2_: *Campylopbacter* Zot protein encoded by Cluster 2 *zot* gene
p-loop NTPase: p-loop containing nucleoside triphosphate hydrolase
