# Supplementary material for: Zonula occludens toxins and their prophages in Campylobacter species
Source: Gut Pathog. 2016 Sep 15;8:43. doi: 10.1186/s13099-016-0125-1 (PMC5025632; doi:10.1186/s13099-016-0125-1)
Supplement: Supplementary file 1 — 10.1186/s13099-016-0125-1 Protein identities between prophages in other Campylobacter species and C. concisus CON_phi2 and CON_phi3. Zot proteins are in bold. Integrase proteins are underlined #Identity: Percentage of identical amino acids (number of identical amino acids divided by number of amino acids in proteins from C. concisus 13826). [file 13099_2016_125_MOESM1_ESM.docx]

**Additional file 1. Protein identities between prophages in other *Campylobacter* species and *C. concisus* CON_phi2 and CON_phi3**

| **Proteins in identified prophages** | | **Proteins in CON_phi2** | **Identity (%)^#^** | **Proteins in CON_phi3** | | **Identity (%)^#^** |
| --- | --- | --- | --- | --- | --- | --- |
| URE_phiZA  (*C. ureolyticus*  DSM 20703) | C512_RS09455 | CCC13826_2082 | 17 | CCC13826_0706 | 18 | |
|  |  | CCC13826_RS07955 |  |  |  | |
|  |  |  |  | CCC13826_0188 |  | |
|  | C512_RS0103955 | CCC13826_1099 | 59 | CCC13826_0189 | 27 | |
|  |  |  |  |  |  | |
|  | C512_RS0103950 | CCC13826_1100 | 27 | CCC13826_0190 | 8 | |
|  | C512_RS0103945 |  |  |  |  | |
|  | C512_RS0103940 |  |  |  |  | |
|  | **C512_RS0103935** | **CCC13826_2276** | **59** | **CCC13826_0191** | 26 | |
|  | C512_RS0103930 | CCC13826_2277 | 66 | CCC13826_0192 | 24 | |
|  | C512_RS0103925 | CCC13826_0183 | 27 | CCC13826_0193 | 16 | |
|  | C512_RS0103920 |  |  |  |  | |
|  | C512_RS0103905 | CCC13826_RS07985 | 67 |  |  | |
|  | C512_RS0103900 | CCC13826_2278 | 67 | CCC13826_0196 | 28 | |
|  | C512_RS0103895 | CCC13826_RS07795 | 70 | CCC13826_0197 | 15 | |
|  | C512_RS0103890 | CCC13826_0164 | 41 | CCC13826_0198 | 32 | |
|  | C512_RS0103885 | CCC13826_2078 | 43 | CCC13826_0199 | 25 | |
|  | C512_RS0103880 |  |  |  |  | |
|  | C512_RS0103875 |  |  |  |  | |
| COR_phiZA  (*C. corcagiensis*  CIT045) | BG71_RS0106465 | CCC13826_2082 | 25 | CCC13826_0706 | 26 | |
|  | BG71_RS0106470 | CCC13826_RS07955 | 10 | CCC13826_0188 |  | |
|  | BG71_RS0106475 | CCC13826_1099 | 62 | CCC13826_0189 | 25 | |
|  | BG71_RS0106480 | CCC13826_1100 | 66 | CCC13826_0190 | 24 | |
|  | **BG71_RS0106485** | **CCC13826_2276** | **56** | **CCC13826_0191** | **27** | |
|  | BG71_RS0106490 | CCC13826_2277 | 52 | CCC13826_0192 | 21 | |
|  | BG71_RS0106495 | CCC13826_0183 | 45 | CCC13826_0193 | 20 | |
|  | BG71_RS0106510 | CCC13826_RS07985 | 65 |  |  | |
|  | BG71_RS0106515 | CCC13826_2278 | 73 | CCC13826_0196 | 33 | |
|  | BG71_RS0106520 | CCC13826_RS07795 | 72 | CCC13826_0197 | 15 | |
|  | BG71_RS0106525 |  |  |  |  | |
|  | BG71_RS0106530 |  |  |  |  | |
|  | BG71_RS0106535 | CCC13826_0164 | 42 | CCC13826_0198 | 30 | |
|  | BG71_RS0106540 | CCC13826_2078 | 23 | CCC13826_0199 | 20 | |
|  | BG71_RS0106545 |  |  |  |  | |
|  | BG71_RS0106550 |  |  |  |  | |
| URE_phiZB  (*C. ureolyticus*  DSM 20703) | C512_RS0100760 | CCC13826_2082 | 42 | CCC13826_0706 | 42 | |
|  |  | CCC13826_RS07955 |  | CCC13826_0188 |  | |
|  | C512_RS0100755 | CCC13826_1099 | 28 | CCC13826_0189 | 25 | |
|  | C512_RS0100750 | CCC13826_1100 | 22 | CCC13826_0190 | 45 | |
|  | **C512_RS0100745** | **CCC13826_2276** | **27** | **CCC13826_0191** | **41** | |
|  | C512_RS0100740 | CCC13826_2277 | 18 | CCC13826_0192 | 25 | |
|  | C512_RS09835 | CCC13826_0183 | 13 | CCC13826_0193 | 20 | |
|  |  | CCC13826_RS07985 |  |  |  | |
|  |  | CCC13826_2278 |  |  |  | |
|  |  | CCC13826_RS07795 |  |  |  | |
|  | C512_RS09350 | CCC13826_2278 | 33 | CCC13826_0196 | 40 | |
|  | C512_RS0100715 | CCC13826_RS07795 | 19 | CCC13826_0197 | 22 | |
|  | C512_RS0100710 | CCC13826_0164 | 24 | CCC13826_0198 | 72 | |
|  | C512_RS0100705 | CCC13826_2078 | 27 | CCC13826_0199 | 21 | |
| GRA_phiZ  (*C. gracilis*  RM3268) | CAMGR0001_2460 | CCC13826_2082 | 24 | CCC13826_0706 | 25 | |
|  | CAMGR0001_2459 | CCC13826_RS07955 | 17 | CCC13826_0188 | 14 | |
|  | CAMGR0001_2458 | CCC13826_1099 | 20 | CCC13826_0189 | 34 | |
|  | CAMGR0001_2457 | CCC13826_1100 | 20 | CCC13826_0190 | 45 | |
|  | **CAMGR0001_2456** | **CCC13826_2276** | **26** | **CCC13826_0191** | **44** | |
|  | CAMGR0001_2455 | CCC13826_2277 | 20 | CCC13826_0192 | 28 | |
|  | CAMGR0001_2454 | CCC13826_0183 | 13 | CCC13826_0193 | 20 | |
|  |  | CCC13826_RS07985 |  |  |  | |
|  | CAMGR0001_2451 | CCC13826_2278 | 23 | CCC13826_0196 | 50 | |
|  | CAMGR0001_2450 | CCC13826_RS07795 | 15 | CCC13826_0197 | 28 | |
|  | CAMGR0001_2449 | CCC13826_0164 | 53 | CCC13826_0198 | 29 | |
|  | CAMGR0001_2448 | CCC13826_2078 | 17 | CCC13826_0199 | 16 | |
|  | CAMGR0001_2447 |  |  |  |  | |
|  | CAMGR0001_2446 |  |  |  |  | |
|  | CAMGR0001_2445 |  |  |  |  | |
| DOYLEI_phiZ  (*C. jejuni* subsp*. doylei*  269.97) | JJD26997_0344 | CCC13826_2082 | 56 | CCC13826_0706 | 56 | |
|  |  | CCC13826_RS07955 |  | CCC13826_0188 |  | |
|  | JJD26997_RS01580 | CCC13826_1099 | 21 | CCC13826_0189 | 38 | |
|  | JJD26997_0347 | CCC13826_1100 | 22 | CCC13826_0190 | 43 | |
|  | **JJD26997_0348** | **CCC13826_2276** | **25** | **CCC13826_0191** | **40** | |
|  | JJD26997_0349 | CCC13826_2277 | 18 | CCC13826_0192 | 26 | |
|  | JJD26997_0350 | CCC13826_0183 | 19 | CCC13826_0193 | 24 | |
|  |  | CCC13826_RS07985 |  |  |  | |
|  | JJD26997_0353 | CCC13826_2278 | 30 | CCC13826_0196 | 36 | |
|  | JJD26997_0354 | CCC13826_RS07795 | 19 | CCC13826_0197 | 47 | |
|  | JJD26997_0355 | CCC13826_0164 |  | CCC13826_0198 | 39 | |
|  | JJD26997_0356 | CCC13826_2078 | 17 | CCC13826_0199 | 18 | |
|  | JJD26997_0357 |  |  |  |  | |
|  | JJD26997_0358 |  |  |  |  | |
| JEJUNI_phiZ  (*C. jejuni* subsp*. jejuni*  60004) | CJE11_RS08075 | CCC13826_2082 | 51 | CCC13826_0706 | 51 | |
|  |  | CCC13826_RS07955 |  | CCC13826_0188 |  | |
|  | CJE11_RS08070 | CCC13826_1099 | 26 | CCC13826_0189 | 45 | |
|  | CJE11_RS08065 | CCC13826_1100 | 22 | CCC13826_0190 | 44 | |
|  | **CJE11_RS08060** | **CCC13826_2276** | **25** | **CCC13826_0191** | **40** | |
|  | CJE11_RS08055 | CCC13826_2277 | 19 | CCC13826_0192 | 25 | |
|  | CJE11_RS08050 | CCC13826_0183 | 6 | CCC13826_0193 | 8 | |
| COR_phiZB  (*C. corcagiensis*  CIT045) | BG71_RS0104635 | CCC13826_2082 | 60 | CCC13826_0706 | 60 | |
|  |  | CCC13826_RS07955 |  | CCC13826_0188 |  | |
|  | BG71_RS0104630 | CCC13826_1099 | 28 | CCC13826_0189 | 25 | |
|  | BG71_RS0104625 | CCC13826_1100 | 20 | CCC13826_0190 | 46 | |
|  | **BG71_RS0104620** | **CCC13826_2276** | **26** | **CCC13826_0191** | **41** | |
|  | BG71_RS0104615 | CCC13826_2277 | 18 | CCC13826_0192 | 25 | |
|  | BG71_RS0104610 | CCC13826_0183 | 16 | CCC13826_0193 | 22 | |
|  |  | CCC13826_RS07985 |  |  |  | |
|  | BG71_RS0104590 | CCC13826_2278 | 29 | CCC13826_0196 | 35 | |
|  |  | CCC13826_RS07795 |  |  |  | |
|  | BG71_RS0104585 |  |  |  |  | |
|  | BG71_RS0104580 |  |  | CCC13826_0197 | 22 | |
|  | BG71_RS0104575 | CCC13826_0164 | 24 | CCC13826_0198 | 72 | |
|  | BG71_RS0104570 | CCC13826_2078 | 27 | CCC13826_0199 | 26 | |
| HYO_phiZ  (*C. hyointestinalis* subsp*. hyointestinalis*  DSM 19053) | CR67_01855 | CCC13826_2082 | 25 | CCC13826_0706 | 25 | |
|  |  | CCC13826_RS07955 |  | CCC13826_0188 |  | |
|  | CR67_01860 | CCC13826_1099 | 28 | CCC13826_0189 | 41 | |
|  | CR67_01865 | CCC13826_1100 | 23 | CCC13826_0190 | 45 | |
|  | **CR67_01870** | **CCC13826_2276** | **26** | **CCC13826_0191** | **43** | |
|  | CR67_01875 | CCC13826_2277 | 22 | CCC13826_0192 | 37 | |
|  | CR67_01880 | CCC13826_0183 | 12 | CCC13826_0193 | 18 | |
|  |  | CCC13826_RS07985 |  |  |  | |
|  | CR67_RS01845 | CCC13826_2278 | 22 | CCC13826_0196 | 38 | |
|  | CR67_01900 | CCC13826_RS07795 | 18 | CCC13826_0197 | 30 | |
|  | CR67_01905 | CCC13826_0164 | 72 | CCC13826_0198 | 25 | |
|  | CR67_01910 | CCC13826_2078 | 19 | CCC13826_0199 | 14 | |
|  | CR67_01915 |  |  |  |  | |
|  | CR67_RS01870 |  |  |  |  | |
| LAW_phiZ  (*C. hyointestinalis*  *subsp. lawsonii*  CCUG 27631) | CHL_RS06780 | CCC13826_2082 | 25 | CCC13826_0706 | 25 | |
|  |  | CCC13826_RS07955 |  |  |  | |
|  |  |  |  | CCC13826_0188 |  | |
|  | CHL_RS06775 | CCC13826_1099 | 24 | CCC13826_0189 | 37 | |
|  | CHL_RS06770 | CCC13826_1100 | 23 | CCC13826_0190 | 45 | |
|  | **CHL_RS06765** | **CCC13826_2276** | **25** | **CCC13826_0191** | **42** | |
|  | CHL_RS06760 | CCC13826_2277 | 24 | CCC13826_0192 | 34 | |
|  | CHL_RS06755 | CCC13826_0183 | 16 | CCC13826_0193 | 22 | |
|  |  | CCC13826_RS07985 |  |  |  | |
|  | CHL_RS06750 | CCC13826_2278 | 25 | CCC13826_0196 | 36 | |
|  | CHL_RS06745 | CCC13826_RS07795 | 18 | CCC13826_0197 | 30 | |
|  | CHL_RS06740 | CCC13826_0164 | 44 | CCC13826_0198 | 25 | |
|  | CHL_RS06735 | CCC13826_2078 | 16 | CCC13826_0199 | 11 | |
|  | CHL_RS06730 |  |  |  |  | |
|  | CHL_RS06725 |  |  |  |  | |
| IGUA_phiZ  (*C. iguaniorum* RM11343) | CIG11343_RS03985 | CCC13826_2082 | 20 | CCC13826_0706 | 22 | |
|  |  | CCC13826_RS07955 |  |  |  | |
|  |  |  |  | CCC13826_0188 |  | |
|  | CIG11343_RS03980 | CCC13826_1099 | 16 |  |  | |
|  | CIG11343_RS03965 |  |  |  |  | |
|  | CIG11343_RS03960 |  |  | CCC13826_0189 | 37 | |
|  | CIG11343_RS03955 | CCC13826_1100 | 23 | CCC13826_0190 | 43 | |
|  | **CIG11343_RS03950** | **CCC13826_2276** | **25** | **CCC13826_0191** | **40** | |
|  | CIG11343_RS03945 | CCC13826_2277 | 22 | CCC13826_0192 | 25 | |
|  | CIG11343_RS03940 | CCC13826_0183 | 14 | CCC13826_0193 | 23 | |
|  |  | CCC13826_RS07985 |  |  |  | |
|  |  |  |  | CCC13826_0194 |  | |
|  |  |  |  | CCC13826_0195 |  | |
|  | CIG11343_RS03935 | CCC13826_2278 | 22 | CCC13826_0196 | 33 | |
|  | CIG11343_RS03930 | CCC13826_RS07795 | 19 | CCC13826_0197 | 27 | |
|  | CIG11343_RS03925 | CCC13826_0164 | 56 | CCC13826_0198 | 25 | |
|  | CIG11343_RS03920 | CCC13826_2078 | 19 | CCC13826_0199 | 15 | |

Zot proteins are in bold. Integrase proteins are underlined ^#^Identity: Percentage of identical amino acids (number of identical amino acids divided by number of amino acids in proteins from *C. concisus* 13826).
