# Supplementary material for: Zonula occludens toxins and their prophages in Campylobacter species
Source: Gut Pathog. 2016 Sep 15;8:43. doi: 10.1186/s13099-016-0125-1 (PMC5025632; doi:10.1186/s13099-016-0125-1)
Supplement: Supplementary file 2 — 10.1186/s13099-016-0125-1 Comparison of Campylobacter prophage proteins with known viral proteins. #Identity: Percentage of identical amino acids (number of identical amino acids divided by number of amino acids in proteins from Campylobacter species). [file 13099_2016_125_MOESM2_ESM.docx]

**Additional file 2. Comparison of *Campylobacter* prophage proteins with known viral proteins**

| **Locus tag** | **Protein name [virus name]** | **Accession** | **Identity (%)^#^** |
| --- | --- | --- | --- |
| **CON_phi2 (*C. concisus* 13826)** | | | |
| CCC13826_2082 | phage integrase family site specific recombinase [Nitratiruptor phage NrS-1] | BAN05310.1 | 19 |
| CCC13826_RS07955 | L protein [Lettuce yellow mottle virus] | YP_002308376.1 | 31 |
| CCC13826_1099 | DNA replication [Enterobacteria phage fiAA91-ss] | YP_008766955.1 | 21 |
| CCC13826_1100 | G IV protein [Enterobacteria phage Ike] | NP_040579.1 | 19 |
| CCC13826_2276 | VSK-tox1 [Vibrio phage VSK] | NP_542361.2 | 10 |
| CCC13826_2277 | No result |  |  |
| CCC13826_0183 | No result |  |  |
| CCC13826_RS07985 | hypothetical protein VPGG_00022 [Vibrio phage VBM1] | YP_007674331.1 | 22 |
| CCC13826_2278 | No result |  |  |
| CCC13826_RS07795 | No result |  |  |
| CCC13826_0164 | gp47 [Burkholderia phage KL3] | YP_004306459.1 | 26 |
| CCC13826_2078 | No result |  |  |
| **URE_phiZA (*C. ureolyticus* DSM 20703)** | | | |
| C512_RS09455 | integrase [Gordonia phage Vendetta] | ANA85584.1 | 25 |
| C512_RS0103955 | replication protein A [Cronobacter phage ESSI-2] | ADX32367.1 | 20 |
| C512_RS0103950 | G IV protein [Enterobacteria phage Ike] | NP_040579.1 | 15 |
| C512_RS0103945 | No result |  |  |
| C512_RS0103940 | No result |  |  |
| C512_RS0103935 | zot-like protein [Vibrio phage VSKK] | AF452449_3 | 19 |
| C512_RS0103930 | No result |  |  |
| C512_RS0103925 | No result |  |  |
| C512_RS0103920 | No result |  |  |
| C512_RS0103905 | No result |  |  |
| C512_RS0103900 | No result |  |  |
| C512_RS0103895 | No result |  |  |
| C512_RS0103890 | No result |  |  |
| C512_RS0103885 | hypothetical protein [Paramecium bursaria Chlorella virus 1] | NP_048602.2 | 10 |
| C512_RS0103880 | hypothetical protein PRJ_Fausto_00125 [Faustovirus] | AIB51838.1 | 14 |
| C512_RS0103875 | No result | ANA85584.1 | 25 |
| **COR_phiZA (*C. corcagiensis* CIT045)** | | | |
| BG71_RS0106465 | Phage integrase family prophage LambdaCh01 [Clostridium phage phiCD211] | YP_009221649.1 | 26 |
| BG71_RS0106470 | No result |  |  |
| BG71_RS0106475 | putative replication initiation protein P12 [Pseudoalteromonas phage PM2] | NP_049896.1 | 18 |
| BG71_RS0106480 | No result |  |  |
| BG71_RS0106485 | zot-like protein [Vibrio phage VSKK] | AAL40840.1 | 21 |
| BG71_RS0106490 | hypothetical protein HAPG_00097 [Halorubrum phage GNf2] | AGF91282.1 | 19 |
| BG71_RS0106495 | No result |  |  |
| BG71_RS0106510 | No result |  |  |
| BG71_RS0106515 | No result |  |  |
| BG71_RS0106520 | No result |  |  |
| BG71_RS0106525 | No result |  |  |
| BG71_RS0106530 | replication-associated protein [Mink circovirus] | YP_009021891.1 | 22 |
| BG71_RS0106535 | hypothetical protein [Enterococcus phage vB_IME197] |  | 34 |
| BG71_RS0106540 | replication associated protein A [Maize streak virus] |  | 20 |
| BG71_RS0106545 | No result |  |  |
| BG71_RS0106550 | No result |  |  |
| **CON_phi3 (*C. concisus* 13826)** | | | |
| CCC13826_0706 | phage integrase family site specific recombinase [Nitratiruptor phage NrS-1] | BAN05310.1 | 32 |
| CCC13826_0188 | transferase [uncultured Mediterranean phage uvMED] | BAR34785.1 | 43 |
| CCC13826_0189 | rep [Haemophilus phage HP2] | NP_536816.1 | 20 |
| CCC13826_0190 | gene IV product [Enterobacteria phage I2-2] | NP_039614.1 | 21 |
| CCC13826_0191 | No result |  |  |
| CCC13826_0192 | No result |  |  |
| CCC13826_0193 | No result |  |  |
| CCC13826_0196 | No result |  |  |
| CCC13826_0197 | No result |  |  |
| CCC13826_0198 | ORF071 [Staphylococcus phage EW] | YP_240188.1 | 28 |
| CCC13826_0199 | No result |  |  |
| **URE_phiZB (*C. ureolyticus* DSM 20703)** | | | |
| C512_RS0100760 | phage integrase family site specific recombinase [Nitratiruptor phage NrS-1] | BAN05310.1 | 29 |
| C512_RS0100755 | replication protein [Escherichia phage pro147] | YP_009207656.1 | 23 |
| C512_RS0100750 | phage morphogenesis protein [Vibrio phage VFJ] | YP_008130282.1 | 23 |
| C512_RS0100745 | morphogenesis related protein [Thermus phage OH3] | BAS49603.1 | 16 |
| C512_RS0100740 | VP6 [Eyach virus] | AAM18362.1 | 20 |
| C512_RS09835 | hypothetical protein [Xanthomonas citri phage CP2] | YP_007392904.1 | 10 |
| C512_RS09350 | hypothetical protein SEA_TRES_12 [Mycobacterium phage Tres] | ALF01297.1 | 32 |
| C512_RS0100715 | putative tail fiber [Staphylococcus phage CNPH82] | YP_950624.1 | 19 |
| C512_RS0100710 | Cro/Cl family transcriptional regulator, partial [Enterococcus phage MSF1] | AIU68541.1 | 27 |
| C512_RS0100705 | No result |  |  |
| **GRA_phiZ (*C. gracilis* RM3268)** | | | |
| CAMGR0001_2460 | putative integrase [Clostridium phage phi3626] | NP_612851.1 | 23 |
| CAMGR0001_2459 | MGF 110-6L [African swine fever virus] | AJL34182.1 | 14 |
| CAMGR0001_2458 | replication protein [Haemophilus phage HP1] | NP_043478.1 | 20 |
| CAMGR0001_2457 | gene IV product [Enterobacteria phage I2-2] | NP_039614.1 | 20 |
| CAMGR0001_2456 | hypothetical protein ps_1666 [Pandoravirus salinus] | YP_008438072.1 | 10 |
| CAMGR0001_2455 | polyprotein orf1ab [Feline coronavirus UU30] | ADO39849.1 | 23 |
| CAMGR0001_2452 | No result |  |  |
| CAMGR0001_2451 | hypothetical protein ATCV1_z340R [Acanthocystis turfacea Chlorella virus 1] | YP_001426821.1 | 19 |
| CAMGR0001_2450 | terminase large subunit [Lactococcus phage 936 group phage PhiL.18] | ALM63951.1 | 19 |
| CAMGR0001_2449 | hypothetical protein Tsac_2863 [Thermoanaerobacterium phage THSA-485A] | YP_006546304.1 | 29 |
| CAMGR0001_2448 | restriction endonuclease [uncultured Mediterranean phage uvMED] | BAR36400.1 | 24 |
| CAMGR0001_2447 | replicase polyprotein, partial [Human coronavirus NL63] | ACA49952.1 | 28 |
| CAMGR0001_2446 | No result |  |  |
| CAMGR0001_2445 | No result |  |  |
| **DOYLEI_phiZ (*C. jejuni* subsp. *doylei* 269.97)** | | | |
| JJD26997_0344 | phage integrase family site specific recombinase [Nitratiruptor phage NrS-1] | BAN05310.1 | 29 |
| JJD26997_0345 | replication protein [Haemophilus phage HP1] | NP_043478.1 | 20 |
| JJD26997_0347 | putative maturation protein [Vibrio phage fs2] | NP_047372.1 | 22 |
| JJD26997_0348 | ORF7 [Ralstonia phage p12J] | NP_932302.2 | 17 |
| JJD26997_0349 | No result |  |  |
| JJD26997_0350 | hypothetical protein pv_121 [Pithovirus sibericum] | YP_009001023.1 | 17 |
| JJD26997_0353 | No result |  |  |
| JJD26997_0354 | No result |  |  |
| JJD26997_0355 | insertion element transposase helix-turn-helix domain protein [Leptospira phage vB_LbrZ_5399-LE1] | AGS80688.1 | 30 |
| JJD26997_0356 | No result |  |  |
| JJD26997_0357 | No result |  |  |
| JJD26997_0358 | No result |  |  |
| **JEJUNI_phiZ (*C. jejuni* subsp. *jejuni* 60004)** | | | |
| CJE11_RS08075 | Phage integrase family prophage LambdaCh01 [Clostridium phage phiCD211] | YP_009221649.1 | 25 |
| CJE11_RS08070 | replication protein [Haemophilus phage HP1] | NP_043478.1 | 18 |
| CJE11_RS08065 | putative maturation protein [Vibrio phage fs2] | NP_047372.1 | 21 |
| CJE11_RS08060 | ORF7 [Ralstonia phage p12J] | NP_932302.2 | 17 |
| CJE11_RS08055 | No result |  |  |
| CJE11_RS08050 | hypothetical protein CPT_Stills103 [Bacillus phage Stills] | YP_009196988.1 | 15 |
| **COR_phiZB (*C. corcagiensis* CIT045)** | | | |
| BG71_RS0104635 | phage integrase family site specific recombinase [Nitratiruptor phage NrS-1] | BAN05310.1 | 34 |
| BG71_RS0104630 | replication protein [Escherichia phage pro147] | YP_009207656.1 | 21 |
| BG71_RS0104625 | phage morphogenesis protein [Vibrio phage VFJ] | YP_008130282.1 | 23 |
| BG71_RS0104620 | morphogenesis related protein [Thermus phage OH3] | BAS49603.1 | 16 |
| BG71_RS0104615 | hypothetical protein F116p41 [Pseudomonas phage F116] | YP_164305.1 | 20 |
| BG71_RS0104610 | No result |  |  |
| BG71_RS0104590 | nonstructural polyprotein [Plautia stali intestine virus] | NP_620555.1 | 25 |
| BG71_RS0104585 | putative long tail fiber protein [uncultured Mediterranean phage uvMED] | BAR33531.1 | 24 |
| BG71_RS0104580 | No result |  |  |
| BG71_RS0104575 | hypothetical protein [Riemerella phage RAP44] | YP_007003634.1 | 30 |
| BG71_RS0104570 | No result |  |  |
| **HYO_phiZ (*C. hyointestinalis* subsp. *hyointestinalis* DSM 19053)** | |  |  |
| CR67_01855 | Phage integrase family prophage LambdaCh01 [Clostridium phage phiCD211] | YP_009221649.1 | 25 |
| CR67_01860 | No result |  |  |
| CR67_01865 | No result |  |  |
| CR67_01870 | ORF7 [Ralstonia phage p12J] | NP_932302.2 | 16 |
| CR67_01875 | No result |  |  |
| CR67_01880 | No result |  |  |
| CR67_RS01845 | hypothetical protein SP15_067 [Bacillus phage SP-15] | AMM44866.1 | 17 |
| CR67_01900 | No result |  |  |
| CR67_01905 | insertion element transposase helix-turn-helix domain protein [Leptospira phage vB_LbrZ_5399-LE1] | AGS80688.1 | 25 |
| CR67_01910 | DNA replication origin-binding helicase [Human herpesvirus 7] | YP_073813.1 | 14 |
| CR67_01915 | No result |  |  |
| **LAW_phiZ (*C. hyointestinalis* subsp. *lawsonii* CCUG 27631)** | | | |
| CHL_RS06780 | integrase [Gordonia phage Vendetta] | ANA85584.1 | 24 |
| CHL_RS06775 | No result |  |  |
| CHL_RS06770 | No result |  |  |
| CHL_RS06765 | ORF7 [Ralstonia phage p12J] | NP_932302.2 | 16 |
| CHL_RS06760 | No result |  |  |
| CHL_RS06755 | m134R [Myxoma virus] | AGU99816.1 | 21 |
| CHL_RS06750 | No result |  |  |
| CHL_RS06745 | No result |  |  |
| CHL_RS06740 | hypothetical protein vBPspSH6_40 [Pseudoalteromonas phage vB_PspS-H6/1] | ANJ65550.1 | 13 |
| CHL_RS06735 | hypothetical protein RB43ORF177c [Enterobacteria phage RB43] | YP_239153.1 | 25 |
| CHL_RS06730 | No result |  |  |
| CHL_RS06725 | CopG-like RHH protein [Sulfolobus spindle-shaped virus 7] | YP_003331503.1 | 16 |
| **IGUA_phiZ (*C. iguaniorum* RM11343)** | | | |
| CIG11343_RS03985 | putative integrase [Gordonia phage GRU3] | AKJ72266.1 | 21 |
| CIG11343_RS03980 | arginosuccinate synthase family protein [Dickeya phage phiDP10.3] | AIM51500.1 | 8 |
| CIG11343_RS03965 | integrase (XerC) [uncultured Mediterranean phage uvMED] | BAR33071.1 | 28 |
| CIG11343_RS03960 | No result |  |  |
| CIG11343_RS03955 | No result |  |  |
| CIG11343_RS03950 | Adenylate kinase and related kinases [uncultured Mediterranean phage uvMED] | BAR35147.1 | 12 |
| CIG11343_RS03945 | No result |  |  |
| CIG11343_RS03940 | latent membrane protein 1 [Human herpesvirus 4] | ADI59967.1 | 7 |
| CIG11343_RS03935 | No result |  |  |
| CIG11343_RS03930 | No result |  |  |
| CIG11343_RS03925 | No result |  |  |
| CIG11343_RS03920 | putative restriction endonuclase [Clostridium phage c-st] | YP_398621.1 | 23 |

^#^Identity: Percentage of identical amino acids (number of identical amino acids divided by number of amino acids in proteins from *Campylobacter* species)
