# Supplementary material for: Zonula occludens toxins and their prophages in Campylobacter species
Source: Gut Pathog. 2016 Sep 15;8:43. doi: 10.1186/s13099-016-0125-1 (PMC5025632; doi:10.1186/s13099-016-0125-1)
Supplement: Supplementary file 3 — 10.1186/s13099-016-0125-1 Comparison of Campylobacter Zot proteins with V. cholerae Zot and N. meningitidis Zot. #Identity: percentage of identical amino acids (number of identical amino acids. divided by number of amino acids of V. cholerae Zot or N. meningitidis Zot). V. cholerae Zot sequence Accession No. is AAF29547. N. meningitidis Zot sequence Accession No. is EJU63554. [file 13099_2016_125_MOESM3_ESM.docx]

**Additional file 3. Comparison of *Campylobacter* Zot proteins with *V. cholerae* Zot and *N. meningitidis* Zot**

| Proteins | Identity with  *V. cholerae* Zot (%)^#^ | Identity with  *N. meningitidis* Zot (%)^#^ |
| --- | --- | --- |
| *C. concisus* Zot2276 | 18.3 | 16.1 |
| *C. ureolyticus* Zot3935 | 17.3 | 17.5 |
| *C. corcagiensis* Zot6485 | 14.5 | 17.2 |
| *C. concisus* Zot0191 | 16.8 | 19.9 |
| *C. ureolyticus* Zot0745 | 15.8 | 18.8 |
| *C. gracilis* Zot2456 | 17.5 | 18.8 |
| *C. jejuni* subsp. *doylei* Zot0348 | 17.0 | 19.9 |
| *C. jejuni* subsp. *jejuni* Zot8060 | 17.0 | 19.9 |
| *C. corcagiensis* Zot4620 | 17.5 | 18.0 |
| *C. hyointestinalis* subsp. *hyointestinalis* Zot1870 | 15.5 | 18.0 |
| *C. hyointestinalis* subsp. *lawsonii* Zot6765 | 16.8 | 17.1 |
| *C. iguaniorum* Zot3950 | 19.2 | 19.5 |

^#^Identity: percentage of identical amino acids (number of identical amino acids divided by number of amino acids of *V. cholerae* Zot or *N. meningitidis* Zot). *V. cholerae* Zot sequence Accession No. is AAF29547. *N. meningitidis* Zot sequence Accession No. is EJU63554
