# Supplementary material for: Zonula occludens toxins and their prophages in Campylobacter species
Source: Gut Pathog. 2016 Sep 15;8:43. doi: 10.1186/s13099-016-0125-1 (PMC5025632; doi:10.1186/s13099-016-0125-1)
Supplement: Supplementary file 4 — 10.1186/s13099-016-0125-1 Comparison of Zot proteins from Campylobacter species, N. meningitidis and V. cholerae. * indicates identical amino acids (shaded in red). : indicates conservative mutations (shaded in blue). .indicates semi-conservative mutations. Transmembrane domains are underlined. Walker A and walker B motifs in the N-terminus of Campylobacter Zot proteins were identified and boxed. Walker A has a sequence of GxxxxGK[S/T], where x is any residue. Walker B motif has a sequence of hhhh[D/E], where h is a hydrophobic residue [25]. [file 13099_2016_125_MOESM4_ESM.docx]

**Walker A motif**

CON_phi2_Zot2276 -MLSLIIGPPRSGKTYKAVHLINDEYELHL--------------KGESKYRFIYTNINGL 45

URE_phiZA_Zot3935 -MLTLLLGPPRSGKTYKAVNDIYEEYLKFK--------------KNENKYRFIYTNIVGL 45

COR_phiZA_Zot6485 -MLSLILGPPRSGKTYKAVKDINDEYKKYL--------------SNTSKYRNIYCNIGGF 45

CON_phi3_Zot0191 -MITYLIGNPGSGKTYYAVFMIYQLFLYEP--KKTFLTK-FVKPKEKPNYSFCYTNINEF 56

URE_phiZB_Zot0745 MAISYITGIPGSGKSYFAVYQIYKEFLEEPK-KKGFLNF-KKQAPKKSKYLFLYTNINQF 58

GRACI_phiZ_Zot2456 -MITYIVGNPGSGKTYYSVFKIYQLFIFKP--KDTFLSK-VIKPEKQKEYLYCYTNINGF 56

DOYLEI_phiZ_Zot0348 MAITYIVGNPGSGKTYFAVNQIYEYFVLPTLPNKRILGFEIKRKLKIFDYLYCYTNINGL 60

JEJUNI_phiZ_Zot8060 MAITYIVGNPGSGKTYFAVNQIYEYFVLPTLPNKRILGFEIKRKLKIFDYLYCYTNINGL 60

COR_phiZB_Zot4620 MAISYITGIPGSGKSYFAVYQIYKEFLESPK-KKGFLNF-KKQEAKKSKYEFLYTNINQF 58

HYO_phiZ_Zot1870 MAIHYIVGNPGSGKSYYGVYILWDKFIKQTKEPKGFLKQFIK-PKVTKTYDIAYTNINEF 59

LAW_phiZ_Zot6765 MAIHYIVGNPGSGKSYYGVYILWDKFIKQTKEPKGFLKQFIK-PKVTKIYDIAYTNINEF 59

IGUA_phiZ_Zot3950 MSIHYIIGNPGSGKTFYGVNVLYESFIKKPKS--NLLTKFIKSNDEVKKYDIAYTNINQF 58

N.meningitidis_Zot -MIYLFTGNMGTGKTSRVVSMILNNE-------DGLFKM-KLEDGTEVDRPLYFCHIDGL 51

V.choleare_Zot MSIFIHHGAPGSYKTSGALWLRLLPA--------------------IKSGRHIITNVRGL 40

: * : *: : .: :

**Walker B motif**

CON_phi2_Zot2276 KFDHFDGFVKQYDKNDFLTAVSQEYTLSSQYENGFLDNVDNYDEYALKSGIYENYHHCLI 105

URE_phiZA_Zot3935 KFDEFEGFVKPFNKTDFLNATIEESVLNSQHESGFLGDIADYDKYAYEKGIYKNYHHTLI 105

COR_phiZA_Zot6485 KFELFDGFVKKFDKLDFINAVNEENLLNKQYETGFISVGNDYDSYALKNGIYEDYHHCLI 105

CON_phi3_Zot0191 KFELCDKFK-KFDFDEFYLGLRNLYALYKT---G------ATDNEVNEKAKELNLYGCVF 106

URE_phiZB_Zot0745 KFELKDNFI-PFDNIDFNFKLNILYQVYKSVD-G------KDDTLLIEKAKELDLYQVLI 110

GRACI_phiZ_Zot2456 KFDLDNKFI-KFDYEKFYSDLEVLYLLYMD---K------VGDDVLNEKAKELNLHNVLI 106

DOYLEI_phiZ_Zot0348 KFDISDKLI-SFDFDIFYLNLTHLYNLYNQ---G------LNDDELNIKANELNLFKVMF 110

JEJUNI_phiZ_Zot8060 KFDISDKLI-SFDFDIFYLNLTHLYNLYNQ---G------LNDDELNIKANELNLFKVMF 110

COR_phiZB_Zot4620 KFELKDNFI-PFDNTDFNFKLNQLYEVYKSTD-G------KDDALLIEKAKELDLYQVLI 110

HYO_phiZ_Zot1870 KFDKSDKII-PFDFENILSSLTILFNRYKF-E-K------ATDEELIKTAKQLNLLNAIF 110

LAW_phiZ_Zot6765 KFDKSDKII-PFDFENILSSLTILFNRYKF-E-K------ATDEELIKTAKQLNLLNAIF 110

IGUA_phiZ_Zot3950 DFTKSDKIQ-PLVFSEILSKLTLLYNEYKF-N-Q------ASDEVLIQKSKELNLYNALF 109

N.meningitidis_Zot DKRQFKAHE-----------LTE-------------------EQIMSAPLRDVIPEGAVL 81

V.choleare_Zot NLERMAKYL-KMDVSDISIEFID--------------TDHPDGRLTMARFWHWARKDAFL 85

. .:

CON_phi2_Zot2276 VLDEAYNTFTKTFN-------------------DSLGRFLSYHGHFGIDIIFLFQSKRQT 146

URE_phiZA_Zot3935 VLDEAYNTFTKEFN-------------------NSLGRFLSYHGHFGIDVVFLLQSRRQT 146

COR_phiZA_Zot6485 VLDEAYNVFDKKFN-------------------DSLGRFLSYHGHFGIDVVFLLQSKRQT 146

CON_phi3_Zot0191 VLDECHNYFKNQKD-------------------EILVWWLTYHRHLYQDIYLITQDLTLV 147

URE_phiZB_Zot0745 VLDEAHNFLNDKED-------------------EVLKWWLTYHRHLYQDIILITQDFSLI 151

GRACI_phiZ_Zot2456 ILDEAHNFLKAKED-------------------SILVWWLTYHRHLYQDIMLITQDLSLI 147

DOYLEI_phiZ_Zot0348 VIDEAHNFLKNKDD-------------------KILIWWLTYHRHLHQEIIFITQDLSLI 151

JEJUNI_phiZ_Zot8060 VIDEAHNFLKNKDD-------------------KILIWWLTYHRHLHQEIIFITQDLSLI 151

COR_phiZB_Zot4620 VLDEAHNFLNDKED-------------------EVLKWWLTYHRHLYQDIILITQDFSLI 151

HYO_phiZ_Zot1870 VIDEIHNFFNEKEN-------------------EVLIWWLTYHRHLYQELYFITQDLSLV 151

LAW_phiZ_Zot6765 VIDEIHNFFNEKEN-------------------EVLIWWLTYHRHLYQELYFITQDLSLV 151

IGUA_phiZ_Zot3950 VIDEIHNFFNEKEN-------------------EVFIWWLTYHRHLYQELYLITQDLSLV 150

N.meningitidis_Zot IVDEAHYTYPVRAAG---------------RPVPPYIQELTELRHHGHTVILMTQHPSQL 126

V.choleare_Zot FIDECGRIWPPRLTATNLKALDTPPDLVAEDRPESFEVAFDMHRHHGWDICLTTPNIAKV 145

.:** : * : :

CON_phi2_Zot2276 NREYLVHTELMYMAQPSGKRLFSKLFKYKVYSTSSQVNDN--LINS-----ENLKFNQKI 199

URE_phiZA_Zot3935 NREYLVHTELMYMAQPSGKRILSRLFRYKVYLTYLDYQKN--YIKS-----ENLRFNPKI 199

COR_phiZA_Zot6485 NREYLVHTELMYVAQPSGKRLLSKVFRYKVYSTCDPKRDN--LIKT-----DNIKFDSKI 199

CON_phi3_Zot0191 NNEYKRIAEKFYRASDSSRRLFSKKFRYEIYASYRLFKKD--RLEI-----INIPFLQEV 200

URE_phiZB_Zot0745 ATGYKSIAEYFYKAIPAQLRLFKNKFRYQQFSSYKLYDKD--LVNRKG---IHIPILPEV 206

GRACI_phiZ_Zot2456 SNEYKRIAEHFVKAVDSSKRLFKNKFRYMLYGSYKMYQKD--VMQK-----FHVPYLKEV 200

DOYLEI_phiZ_Zot0348 SNEYKRIAEFFYKALDSGKRIYKNSLRYVQFSSYKLYQKD--IVTR-----FSLSLNKEV 204

JEJUNI_phiZ_Zot8060 SNEYKRIAEFFYKALDSGKRIYKNSLRYVQFSSYKLYQKD--IVTR-----FSLSLNKEV 204

COR_phiZB_Zot4620 ATGYKSIAEYFYKAIPAQLRLFKNKFRYQQFSSYKLFDKD--LVNRKG---IHIPILPEV 206

HYO_phiZ_Zot1870 NNEYKRIAEFFYRAVDSSKRFFSKKFRYIQYSNYKLYQKD--IIKT-----FHIDFNQEI 204

LAW_phiZ_Zot6765 NNEYKRIAEFFYRAVDSSKRFFSKKFRYIQYSNYKLYQKD--IIKT-----FHIDFNQEI 204

IGUA_phiZ_Zot3950 NSEYKRIAEFFYKAVDSSKRFFSKKFRYIQYSNYKLYQKD--VVRT-----FHVDFSDEC 203

N.meningitidis_Zot DIFVRN-------LVSKHVHLERKAIGMKQYSWYKCVTSLDNPAGVSGVEVASWKPPKEA 179

V.choleare_Zot HNMIREAAEIGYRHFNRATVGLGAKFTLTTHDAANSGQMDSHALTR-----QVKKIPSPI 200

: .

CON_phi2_Zot2276 SNLYSSGSNEIYKSYATKK------ILFLLAFIVFSYVVYKFLEP---KHEP--AQSTKQ 248

URE_phiZA_Zot3935 SNLYNSGSTKIYKSYATGK------IIFLLLIIFISYFGYKFLKP---KPAK--QETIIT 248

COR_phiZA_Zot6485 SEIYNSGSTQIYKSYATGK------IFMLIVLAIILYFGFKFIGP---PKLE--NDKAKK 248

CON_phi3_Zot0191 FDLYHSGQSSNKKSFVRFY------FFLAFLVFIFLLLFFYFVVM---SLFE--TDKPKN 249

URE_phiZB_Zot0745 FALYHSGDKTSTKSFIRQL------IVIGIMIFILLFIGFKFFIN---KVLL--KDVPKN 255

GRACI_phiZ_Zot2456 FNLYHSGQNASQKSFVRKF------LYVSLFLFITLSIYFYFFVK---SFNS--DESA-D 248

DOYLEI_phiZ_Zot0348 FSLYKSGDNKPNKSFFLKI------FTFLLFSILTLIFCFYIFI----SFFK--SDEIKE 252

JEJUNI_phiZ_Zot8060 FSLYKSGDNKPNKSFFLKV------FTFLLFSILTLIFCFYIFI----SFFK--SDEIKE 252

COR_phiZB_Zot4620 FALYHSGDKTSQKSFIRRF------ILIGILIFIVLFIAFKFFIS---NIIL--KDAPKD 255

HYO_phiZ_Zot1870 FNLYHSGQNGLGTSFVKKY------LFISLIIFGFCIVAFAIFVN---SITP---DTPKK 252

LAW_phiZ_Zot6765 FNLYHSGQNGLGTSFVKKY------LFISLIIFGFCIIAFAIFVN---SITP---DTPKK 252

IGUA_phiZ_Zot3950 FNLYHSGKNGVGSSFVKKY------LLLSLMIAIITAIFFSIFVL---YMTP---DIPEN 251

N.meningitidis_Zot FKYYKSASQHQK--FKKKVP-----WAVWALIAIIGFVGWKSFGIFKVYSKA--TDSRIE 230

V.choleare_Zot FKMYASTTTGKARDTMAGTALWKDRKILFLFGMVFLMFSYSFYGLHDNPIFTGGNDATIE 260

* * . . : :

CON_phi2_Zot2276 ETRFVDLNA--SD--SKNIKAI-----------------SNDADKSDINTTIFND----N 283

URE_phiZA_Zot3935 DERFKDINR--TNQDIKEPQLI-----------------QNSDLNLDLNTTIFND----K 285

COR_phiZA_Zot6485 DEFISEIYV--SDKN--QTLEI-----------------SNYKETIKDENLLLNE----R 283

CON_phi3_Zot0191 ENLPIE-------NKFPAPVSE--QP---KN-SS---LFFDD--KKPKNNNIDLP----E 287

URE_phiZB_Zot0745 EPAISDQQTDLSTNDFLKPVEK--NQ----------------------DLNFESK----Y 287

GRACI_phiZ_Zot2456 SSAPAP------DTQSNQPIET--AS---GNSTK---ALFNA--SNPN--PNEPP----I 286

DOYLEI_phiZ_Zot0348 NNISKESNINLNLNISPNTIKD--KS---ENNLFSNLELIDGSLKDLPLKNVDIN----N 303

JEJUNI_phiZ_Zot8060 NNISKESNINLNLNISPNTIKD--KS---ENNLFSNLELIDGSLKDLPLKNVDIN----N 303

COR_phiZB_Zot4620 NAIKIDEKTEISNNEFLNSVNI---T----------------------DQPYKNR----Y 286

HYO_phiZ_Zot1870 DIQNSNI-Q--NTT--ELPIAK--------NNTF-----------GQISKKINTS----E 284

LAW_phiZ_Zot6765 DIQNSNI-Q--NTTDTAFPITK--------NNTF-----------GQISKKINTS----E 286

IGUA_phiZ_Zot3950 KP----I-Q--DFNSTSKPI-------------------------NKPTIKINTD----D 275

N.meningitidis_Zot QEAQKESVV---QTMTEQPASSEEMPLKNSDNLKP-EDFVPTLPEKPESKPIYNTVRQVK 286

V.choleare_Zot SEQS-------------EPQSK--ATV--GNAVG--------------SKAVAPA---SF 286

.

CON_phi2_Zot2276 KIYLRITCFPSG---CKFRNY----AIDLSLDSF----LELLSSSNC------------- 319

URE_phiZA_Zot3935 RTYLKITCYSHF---CKFRNY----SLDLSLNSF----LELISSFDC------------- 321

COR_phiZA_Zot6485 RIYEKITCFPSS---CKFRSY----SLNLTLDSF----LLLLADSKC------------- 319

CON_phi3_Zot0191 IYIYDITCLNNN---CHFSDD----YHLYPLSLI----TYISSTHTP------------- 323

URE_phiZB_Zot0745 NFVYVFYCLKGY---CNLKDE----KEFYPHDIV----SNIVLSSDP------------- 323

GRACI_phiZ_Zot2456 GYIYQIYCFYDR---CSIQNG---TYDHFDQRYL----NFIFLRSPP------------- 323

DOYLEI_phiZ_Zot0348 SSVYKILCIDTT---CHIDDK-NQNFMHFPLEYF----HFILNEFPP------------- 342

JEJUNI_phiZ_Zot8060 SSVYKILCIDTT---CHIDDK-NQNFMHFPLEYF----HFILNEFPP------------- 342

COR_phiZB_Zot4620 KFTYQFYCIKGY---CNLKGE----KEFLPYDIV----SNIVIDSNP------------- 322

HYO_phiZ_Zot1870 IFYYEINCINLT---CSFPNS----NDKFDKRAI----KFLLNQTEI------------- 320

LAW_phiZ_Zot6765 IFYYEINCINLT---CSFPNS----NDKFDKRAI----KFLLNQTEI------------- 322

IGUA_phiZ_Zot3950 LFFYQIECVFDD---CHFLNS----DQIYDKKII----KFLLNKTEI------------- 311

N.meningitidis_Zot TFEQIAGCIDGGKSDCTCYSN-----QGTPLKEI----TKIM------------------ 319

V.choleare_Zot GFCIGRLCVQDGFVTVGDERYRLVDNLDIPYRGLWATGHHIYKDTLTVFFETESGSVPTE 346

* . :

CON_phi2_Zot2276 ----HIFLHDKKSGNYIDYFVSCNS--------EFERVLKGLE--NSSQRVC-----NE- 359

URE_phiZA_Zot3935 ----YIFLKDEKSANYADYYLSCPL--------DFSKVVSNIN--D-LQEIC-----DE- 360

COR_phiZA_Zot6485 ----SIVLTDKKSSNYIDYYVSCPA--------EFIGFLSKFS--GDDNFYKGSQ--NEN 363

CON_phi3_Zot0191 ----LYFYFEPKSHELVKYYYVFDK--------PVFQNLQKNN--------KGVS--DE- 360

URE_phiZB_Zot0745 ----VYAKEISSFKNMQIYVYVFKD--------PVFDFLKTKK--------GVSE--NEK 361

GRACI_phiZ_Zot2456 ----KFNVRSFKGKGITYFFVGFDK--------PVFDNLKKEE--------LNEK--SS- 360

DOYLEI_phiZ_Zot0348 ----IYHYKNKVNKGYQH-FIIFNF--------EVFNNLKKGV--------LKNE--KDT 379

JEJUNI_phiZ_Zot8060 ----IYHYKNKVNKGYQH-FIIFNF--------EVFNNLKKGV--------LKNE--KNT 379

COR_phiZB_Zot4620 ----VYAKEVSSFKDMQIYIYVFEN--------PVFDFLKTNL--------QGVS--DEK 360

HYO_phiZ_Zot1870 ----LYETKKYNISNVETSIYFLKD--------DVFKILNIKFN-DK----GNTD--EKD 361

LAW_phiZ_Zot6765 ----LYETKKYNISNVETSIYFLKD--------DVFKILNIKFN-DK----GNTD--EKD 363

IGUA_phiZ_Zot3950 ----VYQSIKYRAENLETISYFIKD--------DVFKVLNIKFR-LSYEDKKGLT--DEK 356

N.meningitidis_Zot ----------------------CKE--------YVKNGLPFNPYKDEQQRTEQVEQSAKA 349

V.choleare_Zot LFSSSYRYKVLPLPDFNHF-VVFDTFAAQALWVEVRRGLPVKK--------E-----KE- 391

. : .

CON_phi2_Zot2276 KSPQTDSSSMFPTHK---- 374

URE_phiZA_Zot3935 NKGSFNTFSFK-------- 371

COR_phiZA_Zot6485 YTKNYNSFDFR-------- 374

CON_phi3_Zot0191 -KFNQIPNSSVPAIK---- 374

URE_phiZB_Zot0745 DSFNNSTFNNFKL------ 374

GRACI_phiZ_Zot2456 -FSSAIYSK---------- 368

DOYLEI_phiZ_Zot0348 SFTRSLF------------ 386

JEJUNI_phiZ_Zot8060 SFTRSLF------------ 386

COR_phiZB_Zot4620 NSFNSSFVD-F-------- 370

HYO_phiZ_Zot1870 NSLFSSFGSNNTSRSNQK- 379

LAW_phiZ_Zot6765 NSLFSSFGSNNTSRSNQK- 381

IGUA_phiZ_Zot3950 TSFNSLFGSDEPKRKPNQK 375

N.meningitidis_Zot DKPQVLVMGGKP------- 361

V.choleare_Zot ---ESIIKSFL-------- 399

**Additional file 4. Comparison of Zot proteins from *Campylobacter* species, *N. meningitidis* and *V. cholerae*.** * indicates identical amino acids (shaded in red). : indicates conservative mutations (shaded in blue). .indicates semi-conservative mutations. Transmembrane domains are underlined. Walker A and walker B motifs in the N-terminus of *Campylobacter* Zot proteins were identified and boxed. Walker A has a sequence of GxxxxGK[S/T], where x is any residue. Walker B motif has a sequence of hhhh[D/E], where h is a hydrophobic residue [25].
